# Comparison of Copper Concentration Between Rejected Renal Grafts and Cancerous Kidneys

**DOI:** 10.1007/s12011-018-1621-6

**Published:** 2019-01-15

**Authors:** Aleksandra Wilk, Barbara Wiszniewska, Anna Rzuchowska, Maciej Romanowski, Jacek Różański, Marcin Słojewski, Kazimierz Ciechanowski, Elżbieta Kalisińska

**Affiliations:** 1 Department of Histology and Embryology, Pomeranian Medical University, Szczecin, Poland; 2 Department of Biology and Medical Parasitology, Pomeranian Medical University, Szczecin, Poland; 3 Department and Clinic of General Surgery and Transplantology, Medical University of Warsaw, Warsaw, Poland; 4 Department of Nephrology, Transplantology and Internal Medicine, Pomeranian Medical University, Szczecin, Poland; 5 Department of Urology and Urological Oncology, Pomeranian Medical University, al. Powstańców Wielkopolskich 72, 70-111 Szczecin, Poland

**Keywords:** Copper, Renal grafts, Cortex, Medulla, Kidney, Pathological kidney

## Abstract

In the body, disorders in the composition and concentration of trace elements, including copper, can lead to the development of various alterations that may result in incorrect functioning of the kidneys. Data on the concentrations of copper in human kidneys are discussed; however, little is known about the concentration of trace elements within rejected renal grafts and kidneys with tumor lesions. The aim of our study was to compare the copper concentration between cancerous kidneys and rejected renal grafts with the division on renal cortex and renal medulla. Material consisted of kidneys from patients hospitalized at the Department of Urology and General Surgery and Transplantation of the Independent Public Clinical Hospital No. 2 at the Pomeranian Medical University in Szczecin, north-western Poland. The study material consisted of kidneys with tumor lesions (*n* = 33), and renal grafts (*n* = 10), obtained from patients belongs to the north-western areas of Poland. The examination was performed using ICP-AES method. Regarding the pathological kidneys, excluding grafts, the concentration of Cu in the renal cortex was 52% higher than in medullary region and the difference between the compared concentrations was statistically confirmed (*p* < 0.05). Taking into account renal grafts, the concentration of Cu in the medulla was slightly lower than in the cortex (less than 3%). In summary, copper in rejected and cancerous kidneys tends to accumulate in higher amount in the renal cortex than medulla, what can be explained by the fact that renal corpuscles, where the first phase of filtration is performed, are located only in the cortical region of the kidney. Furthermore, renal grafts accumulate significantly less copper than kidneys with neoplastic changes, what could have been caused by immunosuppressive medicines used by the graft recipients. The lower copper concentration in renal grafts could be a consequence of the altered immune system, including inflammatory process or/and non-immune mechanisms. Additionally, cancerous and non-cancerous kidneys exhibit different perfusion rate in renal glomeruli, what can finally lead to disparity in chemical elements concentration, including copper.

## Introduction

Copper (Cu) is a common element that makes up an estimated 0.007% of the Earth’s crust. In the natural environment, Cu occurs in for form of sulfides and oxides. It is widely used due to its good electrical and thermal conductivity, with applications in the construction, chemical, and agricultural industries, as well as in wood impregnating agents, dyes, fertilizers, and fungicides [7, 28].

In the body, disorders in the composition and concentration of trace elements, including Cu, can lead to the development of various alterations that may result in incorrect functioning of the kidneys [18, 34, 38]. Cu is found in almost every human cell, with the highest concentration recorded in the liver [2, 3, 14]. The kidneys, however, are extremely sensitive to the effects of xenobiotics, including heavy metals [4, 17, 27]. These organs are responsible for the elimination of toxins and metabolic compounds, maintaining the acid–base and water balance, regulating blood pressure, and producing hormones (erythropoietin, calcitriol) and enzymes (renin) [21, 22, 31, 32]. It is known that the proximal renal tubules are the most susceptible to the toxic effects of metals. Metals toxicity is associated with the excessive formation of reactive oxygen species, leading to oxidative stress, in which Cu plays an important role; it also forms part of antioxidant enzymes, such as superoxide dismutase [10, 19, 33].

Data has been presented on the concentrations of copper in human kidneys; however, many publications do not specify whether these values relate to the cortical or medullar parts of the kidney. It is believed that the concentration of heavy metals depends on the part of the organ [35, 36]. Additionally, various biological and environmental factors affect the accumulation of trace elements. It has been also reported that there is an association between the etiology of the disease and the concentrations of trace elements within kidneys [13, 24, 35, 36]. Our earlier studies have indicated that renal grafts accumulate less cadmium, lead, mercury, and vanadium than the kidneys, with other pathological alterations [35, 36]. No data has been found regarding the comparison of concentration of Cu between rejected renal grafts and cancerous kidneys. Perhaps, immunosuppressive drugs that are used by transplant recipients influence on copper concentration in kidneys. The aim of our study was thus to compare the copper concentration between cancerous kidneys and rejected renal grafts with the division on renal cortex and renal medulla.

## Material and Methods

Research was carried out between 2009 and 2011. Material consisted of kidneys from patients hospitalized at the Department of Urology and General Surgery and Transplantation of the Independent Public Clinical Hospital No. 2 at the Pomeranian Medical University in Szczecin, north-western Poland. The examined organs were obtained from women and men age ranged 21 to 70 years and 28 to 76 years, respectively.

The 43 kidneys were derived from patients following nephrectomy due to the presence of tumors (K, *n* = 33) and from kidney grafts from patients following transplantation due to kidney failure (G, *n* = 10). All the cancerous kidneys were obtained from patients suffering from renal cell carcinoma. The renal grafts were collected from patients who had used immunosuppressive drugs for over 6 years. The study was approved by the Bioethics Committee of the Pomeranian Medical University (Resolution No. KB-0080/61/09).

Kidneys were separated into the cortex and medulla. The discrepancy between the number of cortices and medullary parts of the cancerous kidneys is the consequence of the fact that there was not enough material within one renal medulla to obtain the required weight for further examination (0.5 g). The samples were dried to constant weight at 105 °C for the determination of copper. This procedure was used to determine the water content (gravimetric method). Dried samples were grounded in an agate mortar. The ground samples were divided into doses weighing from 0.5 to 1.0 g. The samples were wet mineralized in a mixture of concentrated HNO_3_ and HClO_4_ (Suprapur Merck®).

The Cu determination was performed using inductively coupled plasma atomic emission spectrophotometry (ICP AES), on a Perkin-Elmer Optima 2000 DV. The correctness of the analysis was controlled by determination of the analyzed metal in reference material of a known concentration (Table 1). The concentration of the metal was expressed as μg/g dry weight (dw). The average percentage of water content in either part of the kidney was similar at about 80%.

**Table 1:** Concentration of Cu in the certified reference materials NIST SRM 1577c bovine liver (in micrograms per gram dry weight (dw) (*RM*, reference material; *RV*, reference value; *OD*, own determination

RM	RV	OD (*n* = 4)	Recovery (%)
8414 Bovine muscle	2.84 ± 0.45 μg/g	2.9 ± 0.07 μg/g	102.11
Dolt 4	31.2 ± 1.1 μg/g	31.8 ± 0.6 μg/g	102.92

Statistical analysis used Stat Soft Statistica 9.0 software and Microsoft Excel 2007. Interquartile range (IQR), median (Med) and the percent coefficient of variation (CV) were established for the concentration of the Cu. To evaluate the compliance of the results with the expected normal distribution, Kolmogorov-Smirnov (K-S) tests with Lilliefors correction were used (*p* < 0.05). In addition, mean concentrations in the corresponding parts of the kidney were compared between the different patient groups. As the data distribution was not consistent with the expected normal distribution, Kruskal-Wallis (K-W) tests and Mann-Whitney *U* tests (M-W *U*; *p* < 0.05) were used.

## Results

Both the K-S test and the K-S test with Lilliefors correction showed no characteristics of normal distribution, and therefore the mean concentration of copper in the samples was compared with the use of a non-parametric M-W *U* test.

The values for the separated parts of kidneys were shown in Table 2. Regarding the pathological kidneys, excluding grafts, the concentration of Cu in the renal cortex was 52% higher than in medullary region and the difference between the compared concentrations was statistically confirmed (*p* < 0.05) (Table 3). Taking into account renal grafts, the concentration of Cu in the medulla was slightly lower than in the cortex (less than 3%).

**Table 2:** Concentration of copper (in μg/g dry weight) in the kidneys from all patients (K, kidneys with tumors; G, renal grafts; AM, arithmetic mean; SD, standard deviation; Med, median; CV, coefficient of variation in %)

Parameter	K		G	
	Cortex (*n* = 33)	Medulla (*n* = 32)	Cortex (*n* = 10)	Medulla (*n* = 10)
Min-Max	0.26–21.10	0.47–18.21	2.83–19.32	3.39–15.84
Med	13.07	8.62	4.73	4.61
AM ± SD	12.27 ± 4.94	9.67 ± 4.32	6.33 ± 4.82	5.63 ± 3.66
CV	40.27	44.67	76.17	64.97

**Table 3:** The results of the Mann-Whiney *U* test (M-W) used to investigate differences between copper concentrations (μg/g dw) in individual groups of kidneys samples (K, kidneys with tumors; G, renal grafts)

Part of kidney	Parameter	K	G	M-W U Test K vs G
Cortex	*n* Med	33 13.07	10 4.73	*p* < 0,01
Medulla	*n* Med	32 8.62	10 4.61	*p* < 0,01
M-W *U* Test cortex vs medulla	p	0.04	NS	

**Fig. 1:**
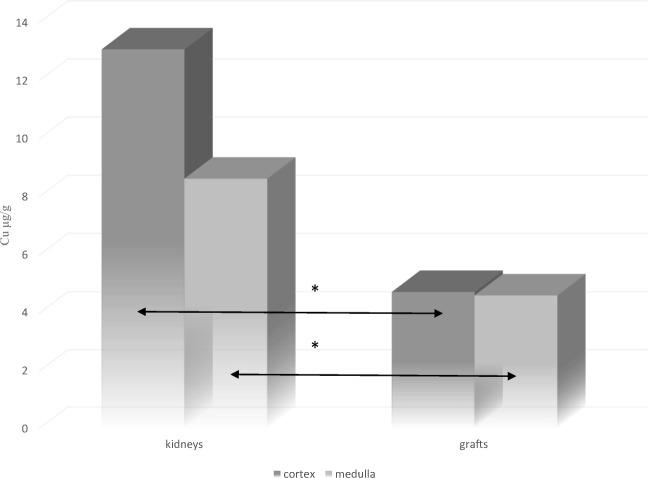
Comparison of Cu concentration between pathologically altered kidneys and rejected renal grafts;*p < 0.01

Comparison of Cu concentrations between cancerous kidneys and renal grafts, showed that both in the cortex and the medulla, the concentration of this metal significantly decreased in the rejected grafts (*p* < 0.05) (Table 3, Fig. 1).

## Discussion

Studies on copper have been conducted for many years [1, 5, 11, 30]. However, little data is available, especially on human pathologically altered kidneys, including renal grafts. The available human studies concern the entire kidney or the renal cortex, and do not specifically take the medullary region into consideration (Table 4). Most articles in the literature refer to animal studies [16, 20, 40].

**Table 4:** Comparison of Cu concentration in humans kidneys in other authors (Med, median; AM, average mean ± SD, standard deviaton)

Material		*n*	Cu concentration (μg/g d.w.)		References
			Med	AM±SD	
Studies based on separated parts of kidneys					
Pathologically altered kidneys (Northern-East Poland)	Cortex	33	13.07	12.27 ± 4.94	Own studies
	Medulla	32	8.62	9.67 ± 4.32	
Renal grafts (Northern-East Poland)	Cortex	10	4.73	6.33 ± 4.82	
	Medulla	10	4.61	5.63 ± 3.66	
Kidneys form autopsy, without pathological alterations (Holand)	Cortex	123	11.50	11.90 ± 3.3	[1]
	Medulla	39	8.50	8.95 ± 2.60	
Studies based only on renal cortex					
Kidneys form autopsy, without pathological alterations (central Poland)	cortex	70		11.31*	[8, 9]
Kidneys form autopsy, without pathological alterations (Upper Silesia, Poland)	cortex	75		10.37*	[8, 9]
Kidneys form autopsy, without pathological alterations (Andalusia, Spain)	cortex	76	21.17*	22.35 ± 11.27*	[26]
Studies based on whole kidney, without division on cortex and medulla					
Kidneys form autopsy, without pathological alterations (London, England), children in different age		1	11.55*		[29]
		1	10.42*		
		1	6.13*		
		1	6.60*		
Kidneys form autopsy, without pathological alterations			13.20		[15]
Kidneys form autopsy, without pathological alterations(South Korea)		83		2.69 ± 1.65	[39]
Kidneys form autopsy, without pathological alterations			14.9		[6]
Kidneys form autopsy, without pathological alterations(South Poland)		76	8.96	10.14 ± 4.24*	[25]

*The original values were given in a wet mass, the dry mass was calculated taking into account the concentration of 78.8% of the water in the kidney (own data)

Determining the concentrations of trace elements in separate parts of the kidney is justified by the tendency for them to accumulate to vary in the cortex and medulla. This occurs due to the differing blood flows in the renal cortex and renal medulla. It is thought that as much as 5–10 times more blood is transported in the cortical renal tissue than in the renal medulla, by volume [12]. Renal corpuscles, where the first phase of filtration is performed, are located only in the cortical region of the kidney. Comparing our results with analogous data presented by other authors, it should be noted that differences in Cu concentration may be caused by different research procedures, different groups of patients, and many other factors that have influence on its concentration.

In kidneys obtained by nephrectomy affected by renal tumors, statistically significant higher Cu concentration was found in the renal cortex than in the medulla. This confirms the greater tendency of the renal cortex to accumulate this trace element, which was also observed by Aalbers et al. (1985): the median concentration of Cu in the renal cortex was 35% higher than in the medulla. However, our data showed higher concentrations of Cu in the renal cortex than in the kidneys studied by Aalbers et al. (1985), at 13.1 μg/g d.w. as compared to 11.5 μg/g d.w. It is possible that other environmental factors affected the Cu concentration in the renal tissue. Considering the renal cortex, our results were similar to those of other authors, even though we compared our data with a healthy group of kidneys (Table 4). The highest concentration of Cu in the kidney cortex (> 22 μg/g d.w., Table 4) was found by [26] who examined the kidneys of patients from Spain. In contrast, Cu concentration in the kidneys of Koreans was less than 2.7 μg/g d.w. [39] and is about four to five times smaller than the values found for the inhabitants of Europe, except Spain (Table 4). The reason for such large discrepancies between the average Cu concentrations of the kidneys of Koreans and the residents of Europe and North America is unknown, but may be associated with different experimental procedures, different groups of patients, and other factors that could affect the concentration of copper.

Due to the intake of immunosuppressive drugs by transplant recipients, the number of nephrons within transplanted graft gradually decreases, which finally leads to organ rejection. It is associated with the fact that immunosuppressive medicines exhibit toxic properties, including nephrotoxicity [21–23, 37]. Interestingly, our results show that there is a significantly lower tendency for copper to accumulate within renal grafts than in kidneys with neoplastic alterations; the median in renal cortices was 4.73 and 13.07, respectively, whereas in the renal medullary regions these values were 4.61 and 8.62, respectively. Similar results have been confirmed in our previous studies that considered cadmium, lead, mercury, and vanadium, where the concentrations of metals were significantly lower in rejected renal grafts [35, 36]. It is likely that immunosuppressive therapy decreases the concentration of heavy metals in some organs, including the kidney. What is more, cancerous kidneys exhibit different metabolism and dynamics of the tumor process, compared to tissue without cancer [24]. The lower copper concentration in renal grafts could be a consequence of the altered immune system, including inflammatory process or/and non-immune mechanisms. Additionally, cancerous and non-cancerous kidneys exhibit different perfusion rate in renal glomeruli, what can finally lead to disparity in chemical elements concentration, including copper [24]. The following research indicates that the concentration of copper evidently decreased within renal grafts, comparing to cancerous kidneys. Furthermore, comparing the copper concentration healthy renal tissue from literature, it can be also claimed, that renal grafts accumulate less copper [8, 9, 26, 39]. However, more research should be performed in this area of nephrotoxicology, including larger number of renal grafts, what is the limitation of the following study. The small number of grafts in our research is due to the fact, that renal graft nephrectomy is infrequent, what makes the study extremely unique. The available literature reports no data on Cu concentrations in rejected renal grafts. The knowledge in this field should be expanded due to improving of transplant recipients lives and prolongation of the proper function of the transplanted organ.

In summary, copper in rejected and cancerous kidneys tends to accumulate in higher amount in the renal cortex than medulla, what can be explained by the fact that renal corpuscles, where the first phase of filtration is performed, are located only in the cortical region of the kidney. Furthermore, renal grafts accumulate significantly less copper than other pathologically altered kidneys, what could have been caused by immunosuppressors used by the graft recipients.
